# Deposition of silver nanoparticles on nanoscroll-supported inorganic solid using incompletely rolled-up kaolinite†

**DOI:** 10.1039/d3ra04383e

**Published:** 2023-09-04

**Authors:** Shingo Machida

**Affiliations:** a Department of Material Science and Technology, Faculty of Advanced Engineering, Tokyo University of Science 6-3-1 Niijuku, Katsushika-ku Tokyo 125-8585 Japan shingo.machida@rs.tus.ac.jp

## Abstract

Nanoscroll-supported platy particles were prepared by incomplete rolling-up of kaolinite layers; when the rolling-up of the kaolinite layer followed by its exfoliation incompletely proceeds, kaolinite nanoscrolls were found at the edge of kaolinite platy particles. To assess the support property of these nanoscroll-supported platy particles, when the deposition of Ag nanoparticles was conducted, these nanoparticles were present on the surface of platy particles and in the tubular interior of nanoscrolls at the edge of platy particles but absent on the surface of ordinal kaolinites, as revealed by X-ray diffraction, X-ray photoelectron spectroscopy, and transmission electron microscopy. These results indicated the successful formation and support property of nanoscroll-supported platy particles.

## Abstract 2

The formation and properties of nanoscroll-supported platy particles were demonstrated by focusing on the incomplete rolling-up of kaolinite layers.

## Graphical abstract

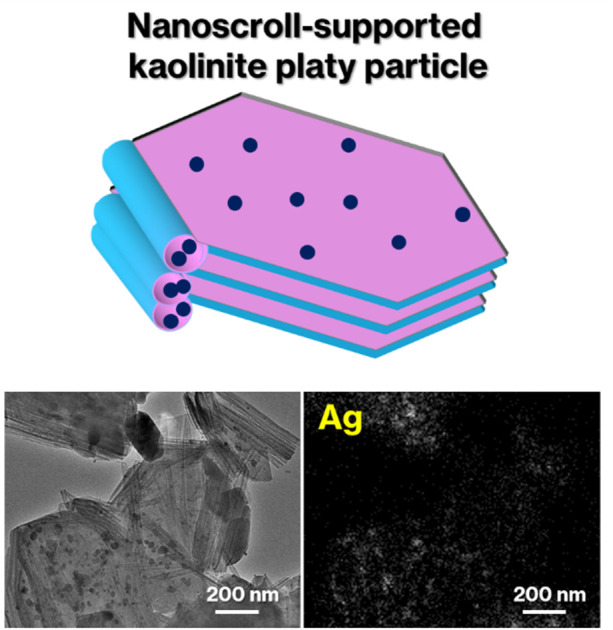

## Introduction

Various fields have widely investigated the fundamental properties and practical applications of nanotubes and nanoscrolls.^1–18^ In addition to their functionalities induced by components, such as carbon and titania, one-dimensional and hollow morphology have been utilized to improve the composite mechanical properties and introduce molecules and nanoparticles to tubular interiors; concerning the latter use, nanotubes and nanoscrolls were used as supports.^1–10^ Layered compounds comprising stacked *ca.* 1 nm layers are also possible fillers of composites and supports for molecules and nanoparticles accommodated into the interlayers, two-dimensional nanospaces.^19–23^ This accommodation requires intercalation, a reaction to insert molecules and ions into the layers to form intercalation compounds.^19–23^ Intercalation reactions can proceed using intercalation compounds as intermediates; thus, multistep reactions can be necessary. However, some layered compounds exhibit low or no intercalation capability.^23,24^ In addition, intercalation with concurrent condensation reactions between molecules and layers cannot be avoided upon the conversion of layered compounds into porous solids, *i.e.*, pillaring.^22^ Notably, nanotubes can be considered dangerous from the viewpoint of cancer risks,^25^ regardless of scientific evidence. Therefore, nanotubes-supported micrometer materials could be promising safety materials for people other than scientists.

In this study, special attention is paid to the preparation of nanotube-supported platy particles. The exfoliation of layered compounds can induce the rolling-up of exfoliated layers.^14^ Kaolinite, a layered clay mineral having the formula of Al_2_Si_2_O_5_(OH)_4_, exhibits the intercalation capability of polar neutral molecules and salts.^14,23,24,26–32^ When specific alkylammonium salts are intercalated between the layers of kaolinite, exfoliation and subsequent rolling-up of kaolinite layers form kaolinite nanoscrolls.^14^ These reactions partially proceed depending on the sorts of alkylammonium salts.^14,27^ Such incomplete proceeding can occur by the deintercalation of molecules from kaolinite layers.^29,30^ The partially rolled-up layers as nanoscrolls are present at the edge of kaolinite platy particles.^29,30^ Thus, the incomplete rolling-up of kaolinite layers is the best method to generate nanoscroll-supported platy particles. Notably, because the intercalation of specific alkylammonium salts developed as an efficient method to exfoliate and roll up kaolinite layers, a method of incomplete rolling-up of the kaolinite layer has never been utilized for material design. In a previous study, the partial rolling-up of kaolinite layers occurred by the deintercalation of octadecyltrimethylammonium chloride (C18TAC) from kaolinite layers upon washing of a kaolinite-C18TAC intercalation compound (Kaol-C18TAC) with a solvent.^29^ Therefore, the support ability of the nanoscroll-supported kaolinite (NS-Kaol) was briefly assessed and compared with the kaolinite. In this trial, because silver nanoparticles were easily obtained by soaking spherical silica particles in a silver nitrate (AgNO_3_) aqueous solution and subsequent calcination,^33^ this method is a suitable candidate to briefly assess the supporting property of NS-Kaol. Thus, the deposition of silver nanoparticles on the present specimens was conducted.

## Experimental

### Materials

The kaolinite used in this study was obtained from the Source Clays Repository of the Clay Material Society (KGa-1b, *i.e.*, well-crystallized Georgia Kaolin), which originally contains anatase as an impurity.^34^ The XRD profile for this kaolinite is shown in Fig. S1.† In addition, KGa-1 was light brown in color because of the presence of Fe as an impurity.^34^*N*-Methylformamide (NMF) and C18TAC were obtained from TCI. Methanol, ethanol, and AgNO_3_ aqueous solution (1 mol L^−1^) were obtained from Wako Pure Chem. The AgNO_3_ aqueous solution was adequately diluted and used. All chemicals were used without further purification.

### Sample preparation

NS-Kaol was prepared according to the previous study; methoxy-modified kaolinite (MeO), a organic derivative of kaolinite in which a portion of hydroxyl groups are substituted to methoxy groups, was prepared using a kaolinite-NMF intercalation compound^35^ as an intermediate, and the intercalation and subsequent deintercalation of C18TAC were conducted.^29^ Ethanol was used for washing Kaol-C18TAC for the deintercalation of C18TAC from kaolinite layers.^29^

NS-Kaol (200 mg) was dispersed in 0.1 or 0.01 mol L^−1^ AgNO_3_ aqueous solution. After the dispersions were allowed to stand for a day in the dark, the resulting solids were centrifuged at 4500 rpm for 5 min and dried at 80 °C for a day in the dark. After drying, the resulting solids were calcined at 500 °C for 2 h at a heating rate of 10 °C min^−1^ to give the specimens denoted as 0.1Ag-NS-K-C and 0.01Ag-NS-K-C. For comparison purposes, similar procedures were applied to MeO-Kaol using 0.01 mol L^−1^ AgNO_3_ aqueous solution to obtain the specimen denoted as 0.1Ag-MeO-K-C. In addition, the resulting solid soaked in 0.1 mol L^−1^ solution was washed with an excess amount of water before drying and then calcined at 500 °C for 2 h to generate the specimen denoted as 0.1Ag-NS-K-Wash-C. Furthermore, NS-Kaol underwent 500 °C-calcination to give the specimen denoted as NS-Kaol-C.

### Characterization

The presence of Ag nanoparticles in the specimens was characterized using X-ray diffraction (XRD) (XRD-6100, Shimadzu), X-ray photoelectron spectroscopy (XPS; JPS-9030, JEOL), transmission electron microscopy (TEM), energy-dispersive X-ray spectroscopy (EDX) mapping, high-angle annular dark field (HAADF) images, and secondary electron (SE) images (HF-2200, Hitachi). The morphology of kaolinite specimens were also characterized using TEM, SE, and HAADF images. TEM images were also obtained using a JEM-2100 (JEOL). Before microscopic analyses, the samples were dispersed in ethanol, and the dispersions were cast to a copper grid. The surface area of NS-Kaol was determined from the nitrogen adsorption isotherm (Belsorp MINI, MicrotracBEL) using the Brunauer–Emmett–Teller (BET) method.^36^

## Results and discussion

**Fig. 1:**
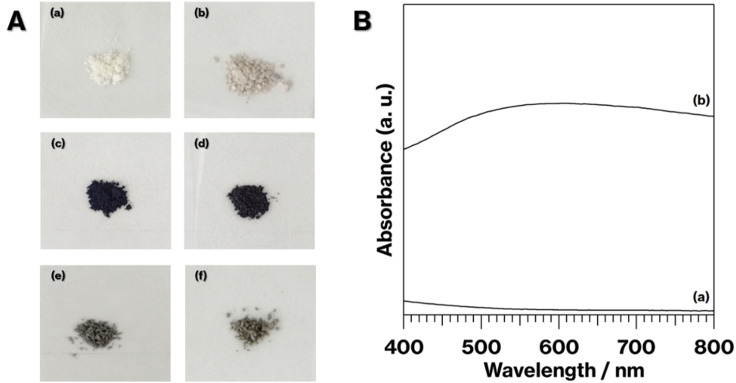
(A) Photographs of (a) NS-Kaol, (b) NS-K-C, (c) 0.1Ag-NS-K-C, (d) 0.01Ag-NS-K-C, (e) 0.1Ag-MeO-Kaol-C, and (f) 0.1Ag-NS-K-Wash-C. (B) Absorption spectra of (a) NS-K-C and (b) 0.1Ag-NS-K-C.

Fig. 1 shows the photographs of the specimens. NS-Kaol was light brown, although NS-Kaol-C was slightly dark compared with NS-Kaol (Fig. 1Aa and Ab). In the previous study, alkylammonium ions electrostatically interacted with kaolinite edges with cation-exchange sites; kaolinite layer surfaces cannot show cation-exchange capability,^27^ whereas kaolinite features the intercalation of polar organic molecules and salts.^14,23,24,26–31^ In addition, after the expansion of kaolinite layers by intercalation, the edge availability to adsorb ions increased; thus, alkylammonium ions were present at kaolinite edges even after the intercalation and subsequent deintercalation of alkylammonium salts.^29^ Furthermore, methoxy groups remained after the intercalation and deintercalation of alkylammonium salts.^29^ In this study, NS-Kaol was prepared by the intercalation and subsequent deintercalation of C18TAC using MeO-Kaol as an intermediate and then calcined at 500 °C. Because plateaus were not observed in the TG curves of kaolinite-alkylammonium salt intercalation compounds and alkylammonium ion-adsorbed kaolinites,^27,29^ the thermal decomposition of organic molecules in kaolinites incompletely proceeded to remain carbon and display a bit black coloration of NS-Kaol-C.

Notably, kaolinite dehydroxylation proceeds at 400–600 °C to form metakaolinite, an amorphous layered aluminosilicate (Al_2_O_3_·2SiO_2_).^31,32,37^ Such dehydroxylation does not change the hexagonal platy particle of kaolinites.^37^ The calcination of kaolinite nanoscrolls is similar to that of kaolinites.^14^ In previous studies, the expansion of the kaolinite layer induced a decrease in the starting temperature of kaolinite dehydroxylation.^26,27^ Depending on the molecules between kaolinite layers and the degree of kaolinite layer expansion, the degree of kaolinite amorphization differed.^31,32^

**Fig. 2:**
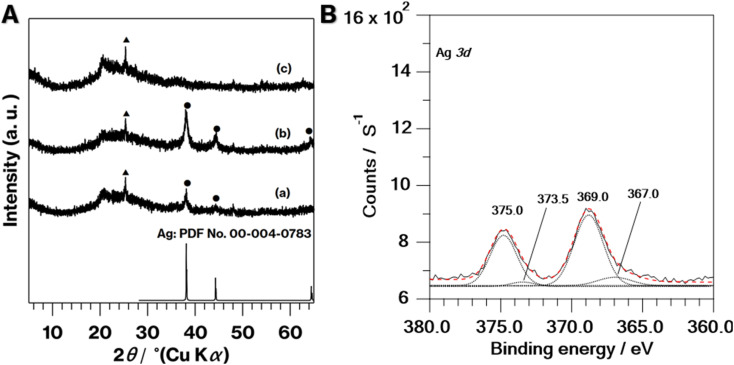
(A) XRD patterns of (a) 0.01Ag-NS-K-C, (b) 0.1Ag-NS-K-C, and (c) NS-Kaol. Closed circles and triangles indicate reflections due to Ag and anatase, respectively. (B) XPS spectrum of 0.1Ag-NS-K-C. Dotted lines are deconvolutional components and red dashed line is the simulated spectrum.

**Fig. 3:**
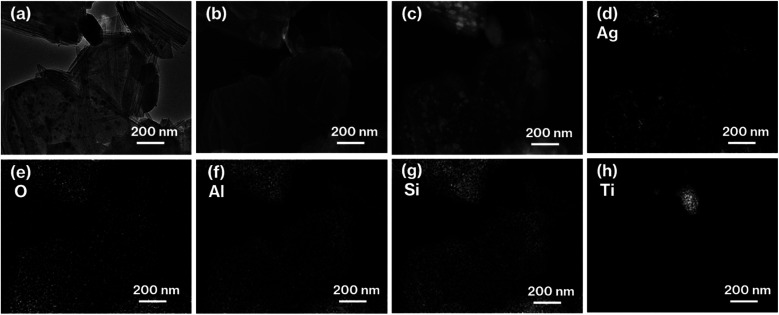
(a) TEM, (b) SE, and (c) HAADF images, as well as EDX mappings of (d) Ag, (e) O, (f) Al, (g) Si, and (h) Ti of 0.1Ag-NS-K-C.

**Fig. 4:**
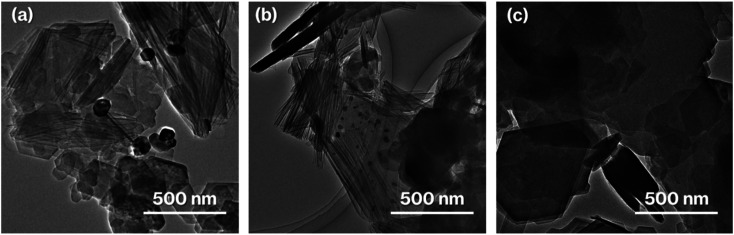
TEM images of (a) NS-Kaol, (b) 0.01Ag-NS-Kaol, and (c) 0.1Ag-MeO-K-C.

The coloration of 0.1Ag-NS-K-C and 0.01Ag-NS-K-C were dark purples, and the coloration of 0.01Ag-NS-K-C was darker than that of 0.01Ag-NS-K-C (Fig. 1Ac and Ad). The absorption spectrum of 0.1Ag-NS-K-C shows a broad absorption peak at around 550 nm, although such absorption is absent in the spectrum of NS-K-C that shows an increase in the intensity in the range of 400–500 nm attributed to the presence of silicate layers (Fig. 1B).^38^ Meanwhile, the XRD patterns of 0.01Ag-NS-K-C and 0.1Ag-NS-K-C show peaks attributed to Ag metal^39^ and a broad profile with kaolinite amorphization;^31,32^ the former Ag peaks were more intense than the latter (Fig. 2A). In addition, the XPS spectrum of 0.1Ag-NS-K-C shows peaks at 375.0 and 369.0 eV attributed to Ad 3d of the Ag metal, which are more intense than those at 373.5 and 367 eV attributed to Ag^+^ (Fig. 2B),^40–42^ indicating that most of the Ag^+^ ions in 0.1Ag-NS-K were reduced under the calcination condition. Based on the peak integrals attributed to Ag 3d (Fig. 2B), Al 2p, and Si 2p of 0.1Ag-NS-K-C (Fig. S2†), the molar ratio of Ag : Al : Si was estimated to be approximately 0.7 : 1 : 1, and the molar ratio of Al : Si is consistent with the formula of kaolinite (Al_2_Si_2_O_5_(OH)_4_). Furthermore, the TEM and HAADF images of 0.1Ag-NS-K-C show ∼50 nm nanoparticles on hexagonal platy particles and in completely and incompletely rolled-up nanoscrolls at the edge of the platy particles (Fig. 3a and c). These nanoparticles are highlighted in the EDX mapping of Ag, but not in that of O (Fig. 3d and e). In previous studies, the Ag nanoparticles in the range of 15–55 nm appeared purple to violet, attributed to plasmonic color.^43–46^ Therefore, the presence of Ag nanoparticles in NS-Kaol is evident in this study. Meanwhile, the presence of nanoscrolls at the edge side of hexagonal platy particles is also evident in the TEM image of NS-K-C (Fig. 4a). This image is well consistent with the TEM and SE images of the present study (Fig. 3a and b) and the SE image of C18TAC-deintercalated kaolinites in a previous study.^29^ In addition, the BET surface area of 32 m^2^ g^−1^ of NS-Kaol, an incomplete rolled-up kaolinite, was smaller than that of the kaolinite nanoscrolls prepared by an efficient rolling up method.^14,47^ Therefore, the formation and the support ability of nanoscroll-supported kaolinites are evident in this study.

Nanoparticles are slightly present in the nanoscrolls at their edges in the TEM image of 0.01Al-NS-K-C (Fig. 4b). In addition, 0.1Ag-MeO-K-C with a small quantity of black coloration (Fig. 1Ae) cannot show the deposition of nanoparticles based on the TEM image (Fig. 4c). Furthermore, a slight black coloration also appeared in 0.1Ag-NS-K-Wash-C. According to the TEM image of 0.1Ag-NS-K-C (Fig. 3a), after a specific concentration of AgNO_3_ aqueous solution was present in NS-Kaol before drying, AgNO_3_ was likely concentrated into mesospaces like nanotubes and then calcined to form Ag nanoparticles; reflections attributed to AgNO_3_ (ref. 39) was present in NS-Kaol soaked in AgNO_3_ aqueous solution before calcination in the XRD pattern (data not shown). Although Ag nanoparticles are present on the platy particles observed in the TEM images of 0.1- and 0.01Ag-NS-K-C, these particles are absent on the calcined MeO-Kaol particles (Fig. 3a, 4b, and 4c). According to the size of Ag particles observed in the TEM images (Fig. 3a and 4b), the mesospaces may likely be generated by overlapping incompletely rolled-up kaolinite layers. Because the kaolinite layer surface does not show cation-exchange capacity, Ag nanoparticles are likely physisorbed on kaolinite surfaces. Notably, kaolinite exhibits an increase in four- and five-fold Al sites, Lewis acid sites, after calcination,^31,32,37^ and the effect of Lewis acid sites on the formation and loading amount of Ag nanoparticles, which strongly relate to the application of Ag nanoparticles,^38–40^ are topic of interests in the future studies. In these trials, the interactions of Ag nanoparticles with inorganic solid surfaces should be compared. Therefore, a further study is required to clarify the surface properties^24^ of nanoscroll-supported kaolinites and the support ability because kaolinites have been used as catalyst supports.^48^

## Conclusions

In summary, this study demonstrated the formation and the support the property of nanoscroll-supported platy particles by focusing on the incomplete rolling-up of kaolinite layers. Given the successful presence of Ag nanoparticles in the tubular interior of nanoscrolls at the edge of the kaolinite in this study and nanoscrolls with varied compositions,^1,9–12^ the present results paved the way for the preparation and utilization of nanoscroll-supported platy particles.

## Conflicts of interest

The author declare no conflict of interest.

## Supplementary Material

RA-013-D3RA04383E-s001
