# Supplementary material for: Deposition of silver nanoparticles on nanoscroll-supported inorganic solid using incompletely rolled-up kaolinite
Source: RSC Adv. 2023 Sep 4;13(38):26430–4. doi: 10.1039/d3ra04383e (PMC10476024; doi:10.1039/d3ra04383e)
Supplement: RA-013-D3RA04383E-s001 [file RA-013-D3RA04383E-s001.pdf]

Electronic Supplementary Material (ESI) for RSC Advances.
This journal is © The Royal Society of Chemistry 2023

Electrical supporting information

Deposition of silver nanoparticles on nanoscroll-supported inorganic solid using incompletely rolled-up kaolinite

Shingo Machida[*]

Department of Material Science and Technology, Faculty of Advanced Engineering, Tokyo University of Science, 6-3-1 Niijuku, Katsushika-ku, Tokyo 125-8585, Japan

[*]E-mail: shingo.machida@rs.tus.ac.jp

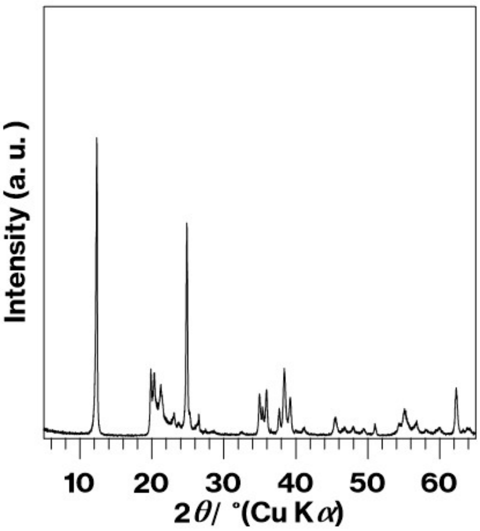

Figure S1. XRD pattern for kaolinite used in this study.

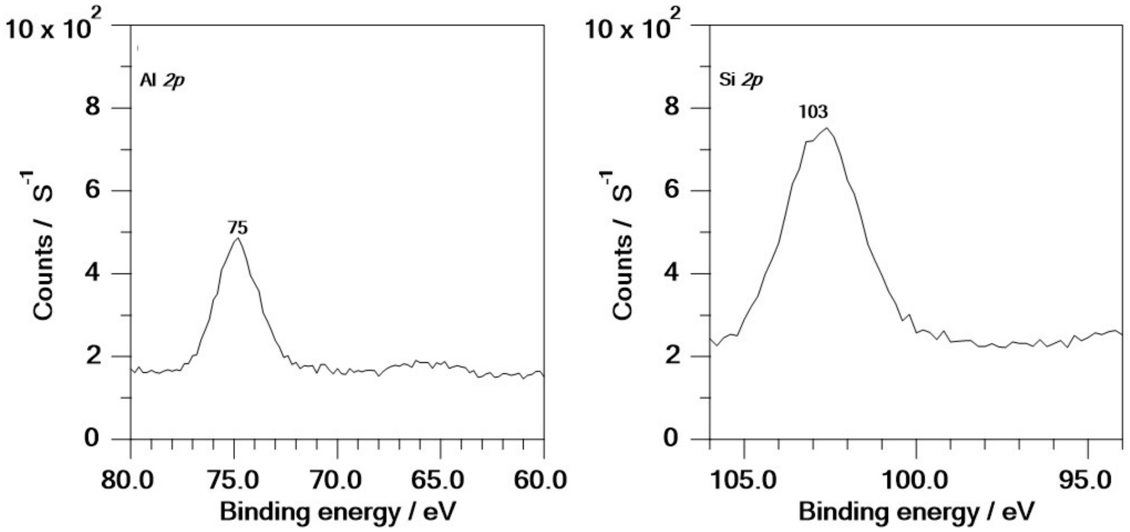

Figure S2 XPS spectra of 0.1Ag-NS-K-C.
